# Supplementary material for: Detecting Rhythmic Gene Expression in Single Cell Transcriptomics
Source: bioRxiv. 2024 Aug 11:2023.12.07.570691. Originally published 2023 Dec 8. Preprint. [Version 2] doi: 10.1101/2023.12.07.570691 (PMC10723455; doi:10.1101/2023.12.07.570691)
Supplement: 1 [file NIHPP2023.12.07.570691v2-supplement-1.pdf]

bioRxiv preprint doi: https://doi.org/10.1101/2023.12.07.570691; this version posted August 11, 2024. The copyright holder for this preprint (which was not certified by peer review) is the author/funder, who has granted bioRxiv a license to display the preprint in perpetuity. It is made available under aCC-BY 4.0 International license.

8 Supplemental material

8.1 Data preprocessing and clustering

Preprocessed data and cell type information was generated as described by Ma et al [23]. Briefly, single cell data from six time points (two experimental replicates, hence twelve in total) were integrated the data on a per time point basis using Seurat. Anchor genes were identified using the FindVariableFeature function, and only those considered to be features at all time points were selected. Prior to integration, the data was first processed using the scTransform function. Raw counts were normalized using the NormalizeData function in Seurat with default parameters. Integrated data were then reduced to 2D using t-SNE, from which cluster identities were assigned. Full preprocessing details may be found in [23], with code and cell-type annotations available from `https://github.com/rosbashlab/scRNA_seq_clock_neurons`. For all of our analysis, we used normalized counts generated by Ma *et al.* to ensure correspondence with results reported in [23]. A copy of the resulting normalized data can be found at `https://bitbucket.org/biocomplexity/singlecell_benchmark`.

8.2 Cell type selection for figures 2, 4

Not all cell clusters were used for Figures 2–4. Our choices are described here.

For Figure 2, we compared how different cycling detection methods perform when considering each cell as a replicate. To ensure the data is compatible with RAIN, JTK-cycle and our subsampling scheme, we combined both replicates so that each time point has at least two cells. To ensure RAIN can be applied with a tractable amount of time, we excluded clusters with more than 120 cells. The selected clusters have sample size ranging from 30 to 117, covering the full range of sample size in this dataset.

For Figure 4, we compared how different cycling methods perform when considering each cell as a replicate and when pseudo-bulk expression profile is generated. We concatenated the two replicates so that the time series spans two periods (corresponding to the actual collection of the data) and selected all cell types with at least two cells at each time point for subsampling.

8.3 Cycling detection

Because the two experimental replicates from Ma et al. were collected consecutively, the data were concatenated to a time series with twelve time points. Consequently, the pseudo-bulk time series also consist of twelve data points, and similarly for the subsampled data (constructed such that each subexperiment has at least one sample from each one of the twelve time points).

We applied four cycling detection methods:

bioRxiv preprint doi: https://doi.org/10.1101/2023.12.07.570691; this version posted August 11, 2024. The copyright holder for this preprint (which was not certified by peer review) is the author/funder, who has granted bioRxiv a license to display the preprint in perpetuity. It is made available under aCC-BY 4.0 International license.

- JTK-cycle is implemented using $JTK\_CYCLEv3.1.R$. The period was set to 6 and the sampling interval was set to 4 to match the experimental procedure. When considering single cells as replicates, we also supplied the number of samples for each time point.
- Harmonic regression is implemented using the harmonicRegression package in R [27] inputting only time and the expression matrix.
- RAIN is implemented in the R package [19]. It takes as input the expression matrix ordered by sampling time and the sampling interval *deltat*, set to 4 to match the experimental design.
- ARSER is implemented using the metacycle package in R [26], whose only required input is the expression matrix and sampling time.

For the purpose of our analysis, a gene is considered to be cycling if its raw p-value is less than 0.05. We also repeat the analysis using FDR–adjusted p-values.

8.4 Derivation of theoretical computational complexity

8.4.1 Mann-Whitney U Statistics

Both JTK cycle and RAIN are non-parametric methods that are built upon the Mann-Whitney U statistics. Let $(X_{11}, \ldots, X_{1m}), \ldots$ $(X_{T1}, \ldots, X_{Tm})$ be a set of T time–associated observations following probability distributions $P_1(x)$, $\ldots$, $P_T(x)$ for timepoints 1–T. For the purpose of deriving computational complexity, we have assumed that each time point contains the same number of cells m without loss of generality. The Mann-Whitney test tests the null hypothesis that $P_i = P_j (i \neq j)$ by computing the U statistic between sample i and sample j as

$$U_{i,j} = \sum_{k=1}^{m} \sum_{l=1}^{m} \mathbb{I}(X_{ik} < X_{jl}) \tag{5}$$

It is easy enough to see that $U_{i,j}$ should have a computational complexity of $\mathcal{O}(m^2)$ and therefore the computational complexity of $\mathbf{U} = (U_{1,2}, \ldots, U_{T-1,T})$ should be approximately $\mathcal{O}(T^2 \times m^2)$. Since in real datasets $T \ll m$ and the number of time points do not change from one cluster to another, our expected observed computational complexity, when changing sample size, should also be $\mathcal{O}(m^2)$.

8.4.2 Jonckheere-Terpstra Test

JTK cycle employs the Jonckheere-Terpstra test, which is an extension of the Mann-Whitney U statistics to the case of having more than two samples. It tests for the presence of monotonic trend with

bioRxiv preprint doi: https://doi.org/10.1101/2023.12.07.570691; this version posted August 11, 2024. The copyright holder for this preprint (which was not certified by peer review) is the author/funder, who has granted bioRxiv a license to display the preprint in perpetuity. It is made available under aCC-BY 4.0 International license.

the test statistics s, defined as

$$s = \sum_{i=1}^{T-1} \sum_{j=i+1}^{T} U_{i,j} \tag{6}$$

Again, it is clear that the computational complexity for s should be $\mathcal{O}(m^2 + T^2)$. When the number of time points is fixed, the computational complexity scales quadratically with sample size.

In JTK cycle, the rising and falling part of the oscillation is tested for each a pre-determined waveform (e.g., sine waves of differing phases), resulting in a complexity of $\mathcal{O}(km^2)$, where k is the number of templates. As k is fixed and $k \ll m$, the complexity will scale approximately $\mathcal{O}(m^2)$.

8.4.3 General Umbrella

To overcome the JTK-cycle's loss of power when considering multiple waveforms, RAIN uses a variation of the general umbrella which tests for the presence of an umbrella shape, which can be formally written as

$$H1 : P_1(x) < P_2(x) < \dots P_t(x) > \cdots > P_T(x) \tag{7}$$

Here, t is a predetermined inflection point. The test statistics of the general umbrella is the sum of two Jonckheere-Terpstra statistics

$$s = \sum_{i=1}^{t-1} \sum_{j=i+1}^{t} U_{i,j} + \sum_{i=t}^{T-1} \sum_{j=i+1}^{T} U_{j,i} \tag{8}$$

RAIN tests for all potential inflection points T, yielding a computational complexity $\mathcal{O}(Tm^2)$. Since T is fixed by the experimental design, the complexity scales with the squared number of cells.

bioRxiv preprint doi: https://doi.org/10.1101/2023.12.07.570691; this version posted August 11, 2024. The copyright holder for this preprint (which was not certified by peer review) is the author/funder, who has granted bioRxiv a license to display the preprint in perpetuity. It is made available under aCC-BY 4.0 International license.

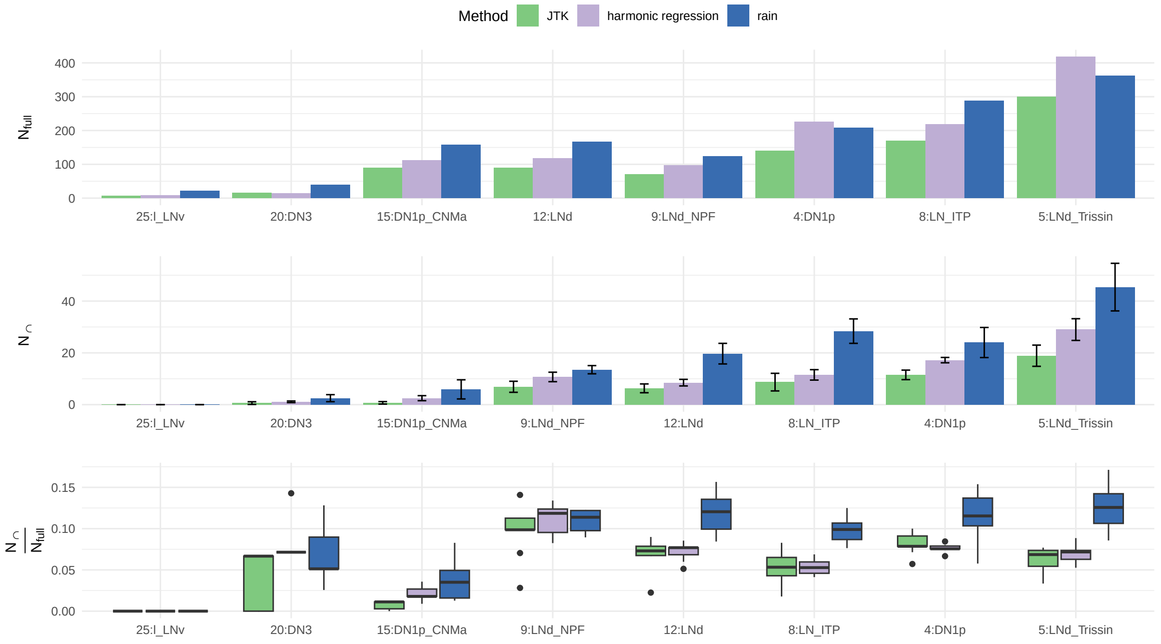

Figure S1: Top: The number of cycling genes detected using all cells, N_{full}. Middle: The average number of cyclers detected in both subsamples across 10 trials, $N_\cap$. Bottom: The ratio of $N_\cap$ to N_{full}, the proportion of consistently–detected sub-sample cyclers relative to those found using all cells. Error bars indicate standard deviation across the 10 subsamplings. A gene is considered cycling if its BH corrected p-value (i.e., FDR) is less than 0.05.

bioRxiv preprint doi: https://doi.org/10.1101/2023.12.07.570691; this version posted August 11, 2024. The copyright holder for this preprint (which was not certified by peer review) is the author/funder, who has granted bioRxiv a license to display the preprint in perpetuity. It is made available under aCC-BY 4.0 International license.

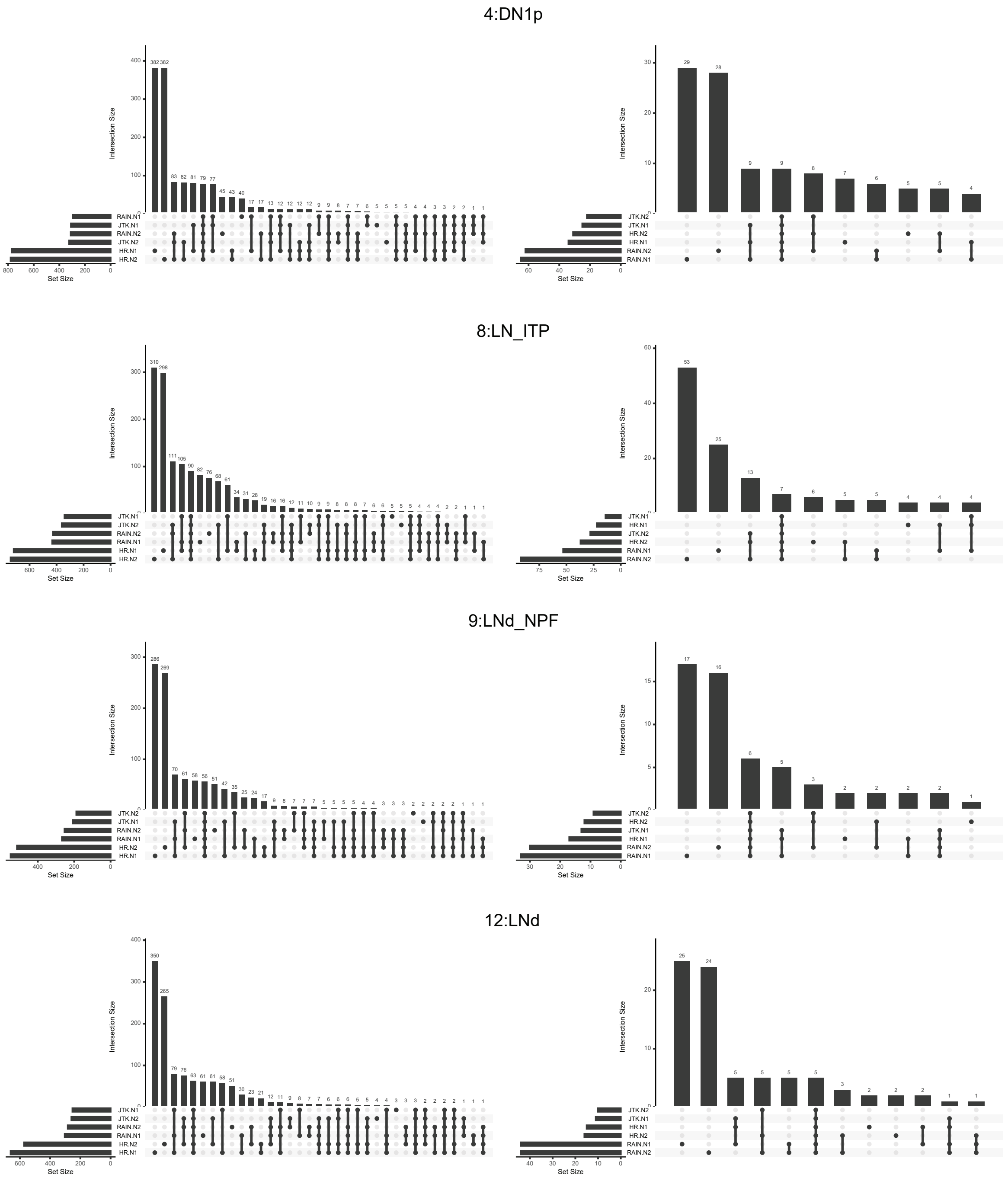

Figure S2: UpSet plots for cluster 4, 8, 9, and 12. The left (right) column uses p-values (adjusted p-values) to identify cycling genes.

bioRxiv preprint doi: https://doi.org/10.1101/2023.12.07.570691; this version posted August 11, 2024. The copyright holder for this preprint (which was not certified by peer review) is the author/funder, who has granted bioRxiv a license to display the preprint in perpetuity. It is made available under aCC-BY 4.0 International license.

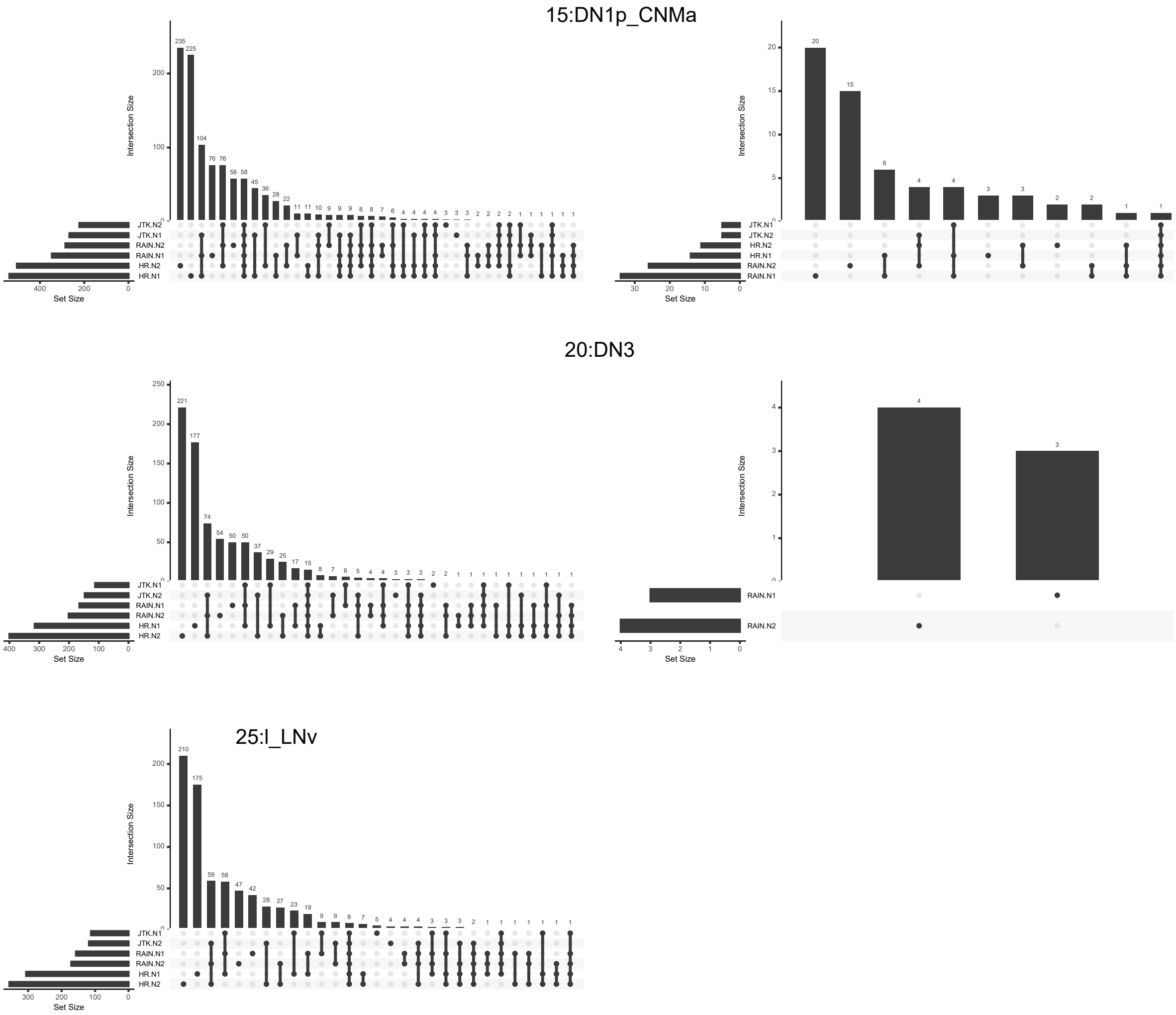

Figure S3: UpSet plots for cluster 15, 20, and 25. The left (right) column uses p-value (adjusted p-values) to identify cycling genes. Right column for cluster 25 is empty for that the average intersection between all sets were less than one.

bioRxiv preprint doi: https://doi.org/10.1101/2023.12.07.570691; this version posted August 11, 2024. The copyright holder for this preprint (which was not certified by peer review) is the author/funder, who has granted bioRxiv a license to display the preprint in perpetuity. It is made available under aCC-BY 4.0 International license.

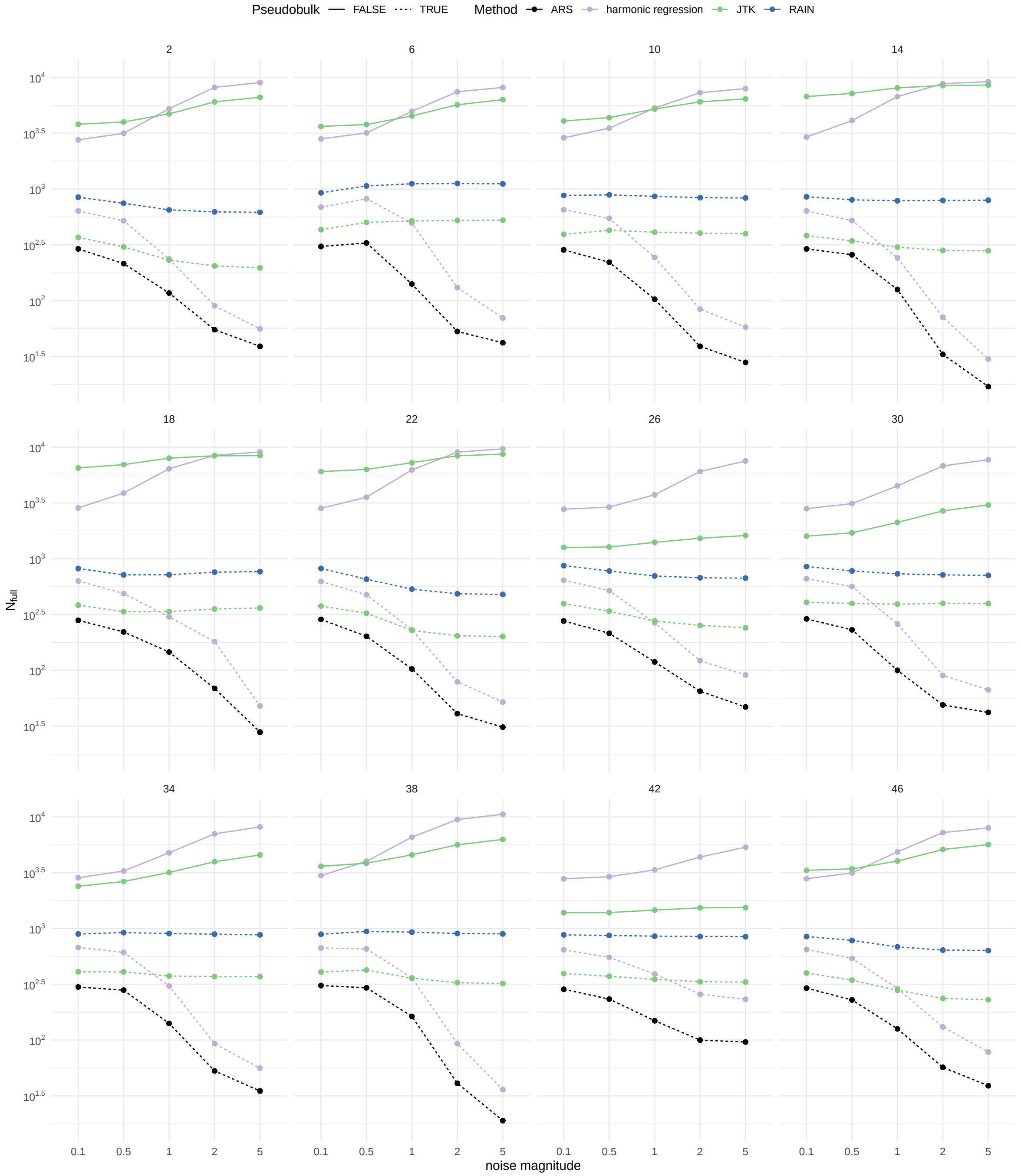

Figure S4: Effect of systematic contamination when using various methods with or without pseudobulking. Each panel shows how the number of detected cyclers N_{full} varies as function of the magnitude of noise injected at a single time-point.

bioRxiv preprint doi: https://doi.org/10.1101/2023.12.07.570691; this version posted August 11, 2024. The copyright holder for this preprint (which was not certified by peer review) is the author/funder, who has granted bioRxiv a license to display the preprint in perpetuity. It is made available under aCC-BY 4.0 International license.

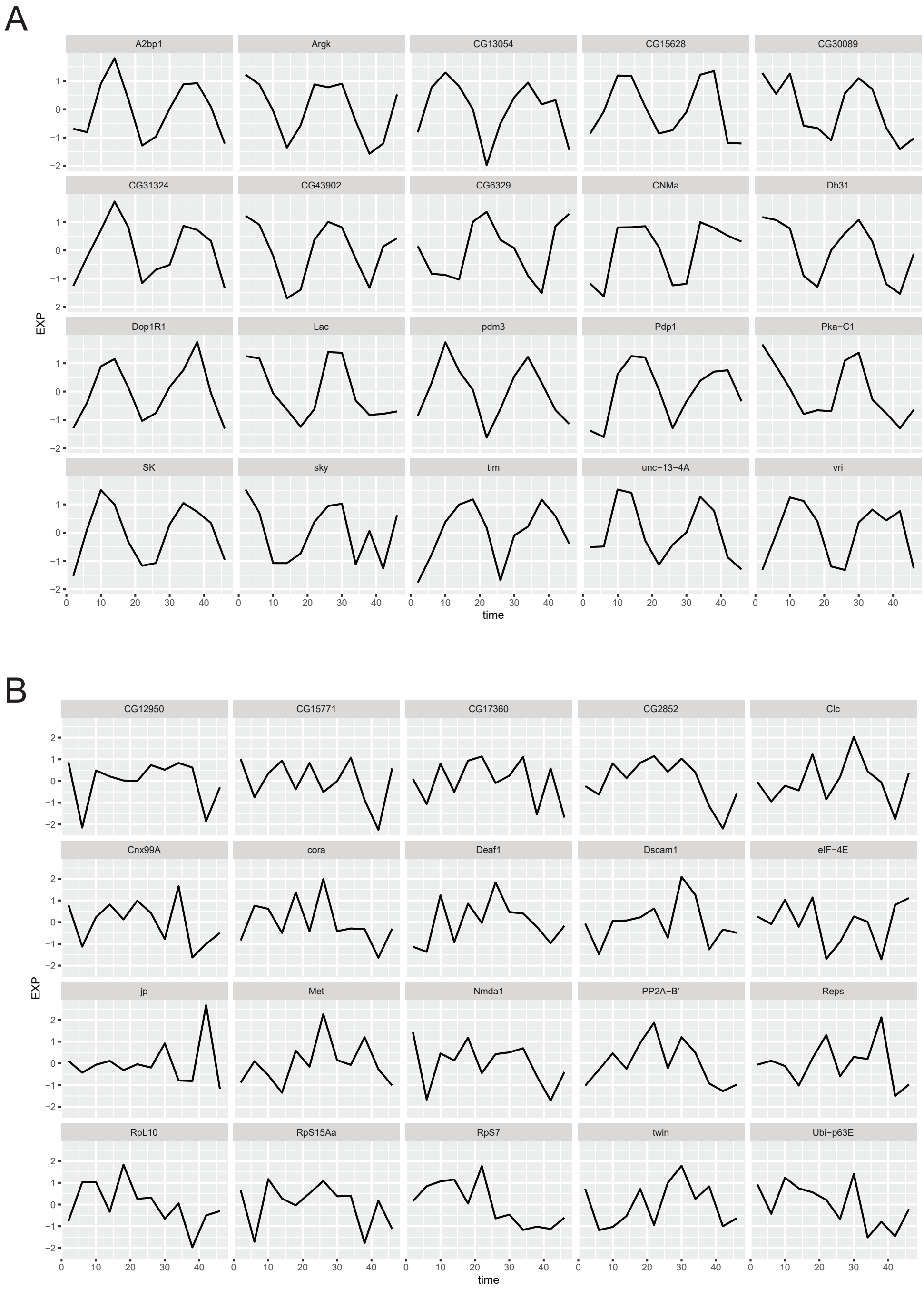

Figure S5: Rhythmic (A) and non-rhythmic (B) genes used for the generation of synthetic data.

bioRxiv preprint doi: https://doi.org/10.1101/2023.12.07.570691; this version posted August 11, 2024. The copyright holder for this preprint (which was not certified by peer review) is the author/funder, who has granted bioRxiv a license to display the preprint in perpetuity. It is made available under aCC-BY 4.0 International license.

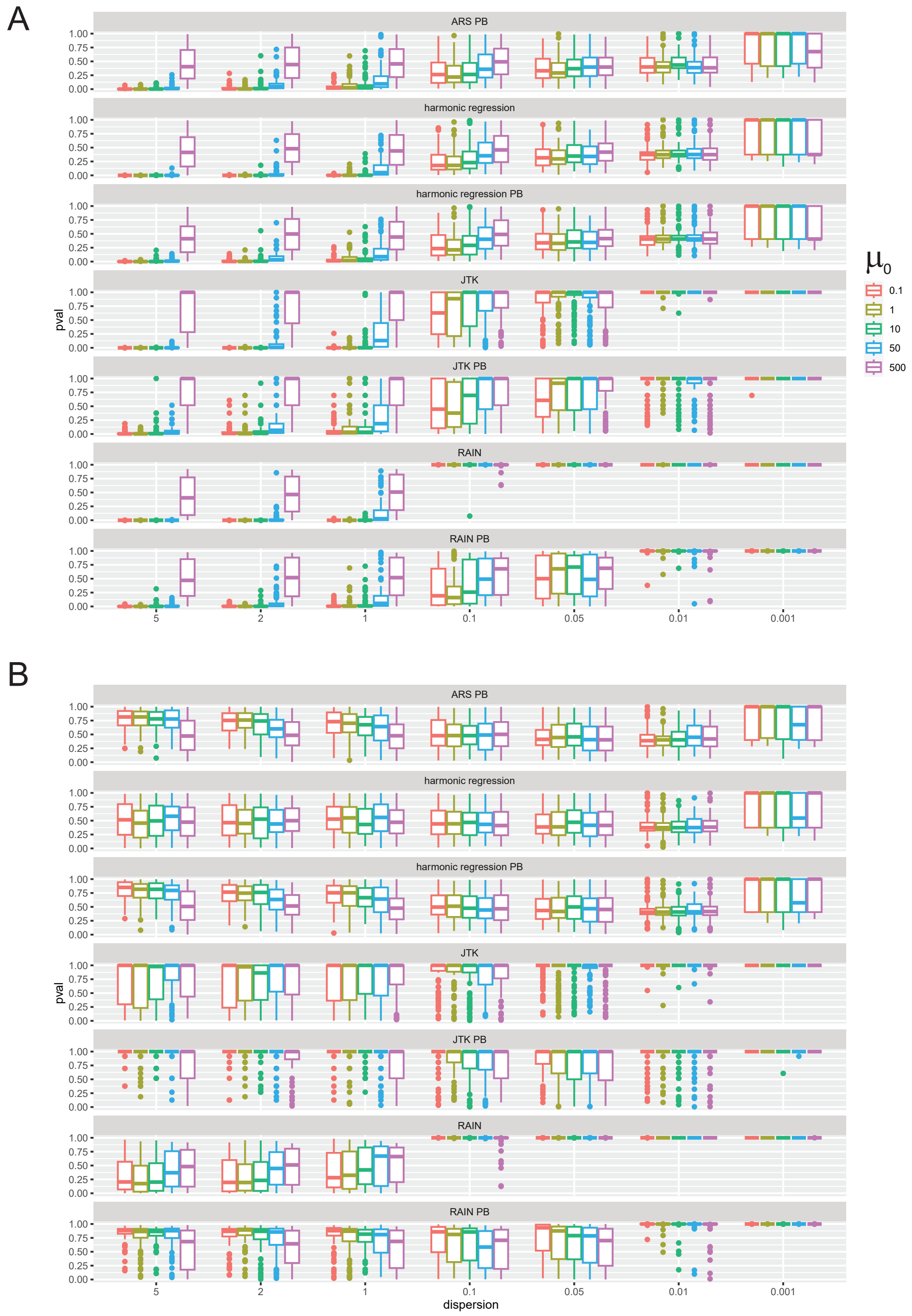

Figure S6: p-value distributions for cycling detection on synthetic rhythmic (A) and non-rhythmic (B) genes under different dispersions and μ_0. Note that for (A) 0 is ideal and for (B) 1 is ideal. Dispersion goes from the largest to smallest so that variance increases from left to right.

bioRxiv preprint doi: https://doi.org/10.1101/2023.12.07.570691; this version posted August 11, 2024. The copyright holder for this preprint (which was not certified by peer review) is the author/funder, who has granted bioRxiv a license to display the preprint in perpetuity. It is made available under aCC-BY 4.0 International license.

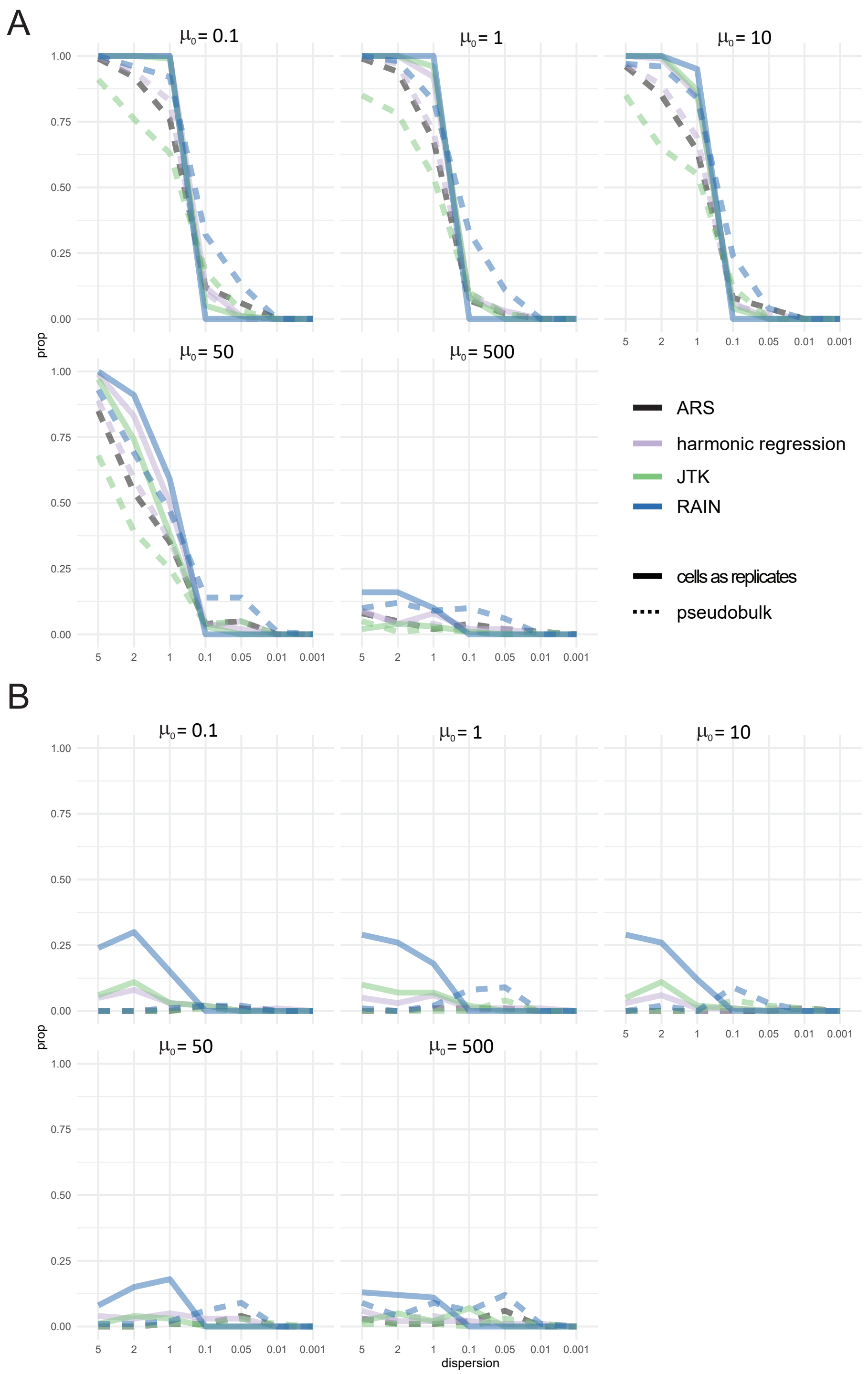

Figure S7: Cycling detection on synthetic rhythmic (A) and non-rhythmic (B) genes under different dispersion value and μ_0. The Y-axis represents the overall proportion of synthetic genes that were considered to be cycling under each tested condition. The number at the top of each graph is the μ_0 level. Note that for (A) 1 is ideal and for (B) 0 is ideal. Dispersion goes from the largest to smallest so that variance increases from left to right.

bioRxiv preprint doi: https://doi.org/10.1101/2023.12.07.570691; this version posted August 11, 2024. The copyright holder for this preprint (which was not certified by peer review) is the author/funder, who has granted bioRxiv a license to display the preprint in perpetuity. It is made available under aCC-BY 4.0 International license.

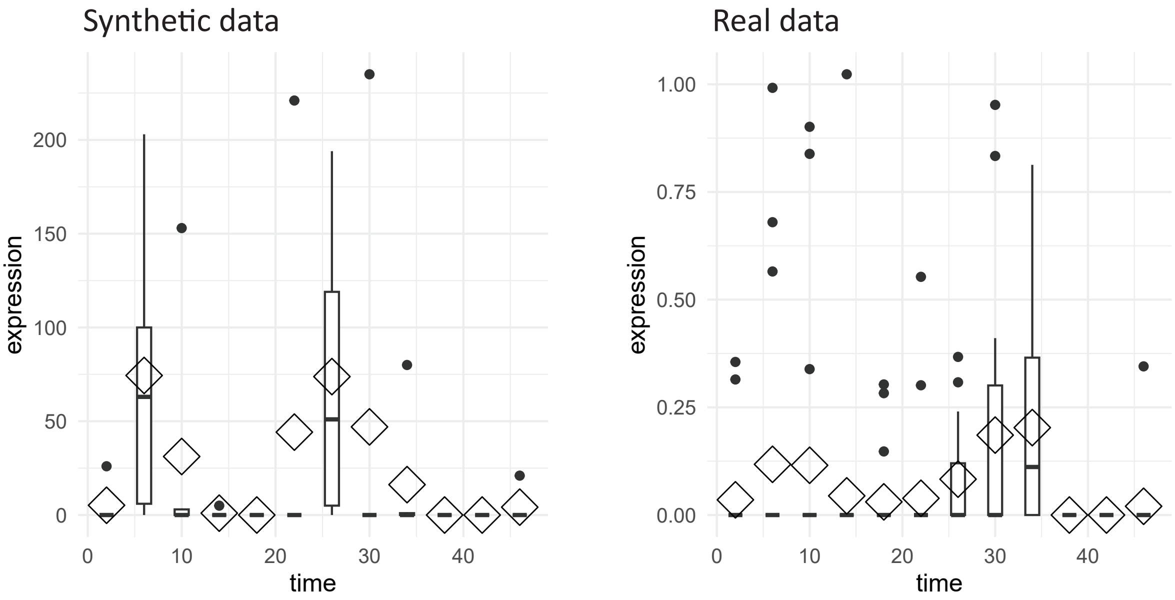

Figure S8: Example synthetic and real data (MsR1) data with oscillations in genes that are mostly unexpressed and are seemingly driven by noise/outliers. Here, diamonds represent means and heavy horizontal lines indicate medians.

bioRxiv preprint doi: https://doi.org/10.1101/2023.12.07.570691; this version posted August 11, 2024. The copyright holder for this preprint (which was not certified by peer review) is the author/funder, who has granted bioRxiv a license to display the preprint in perpetuity. It is made available under aCC-BY 4.0 International license.

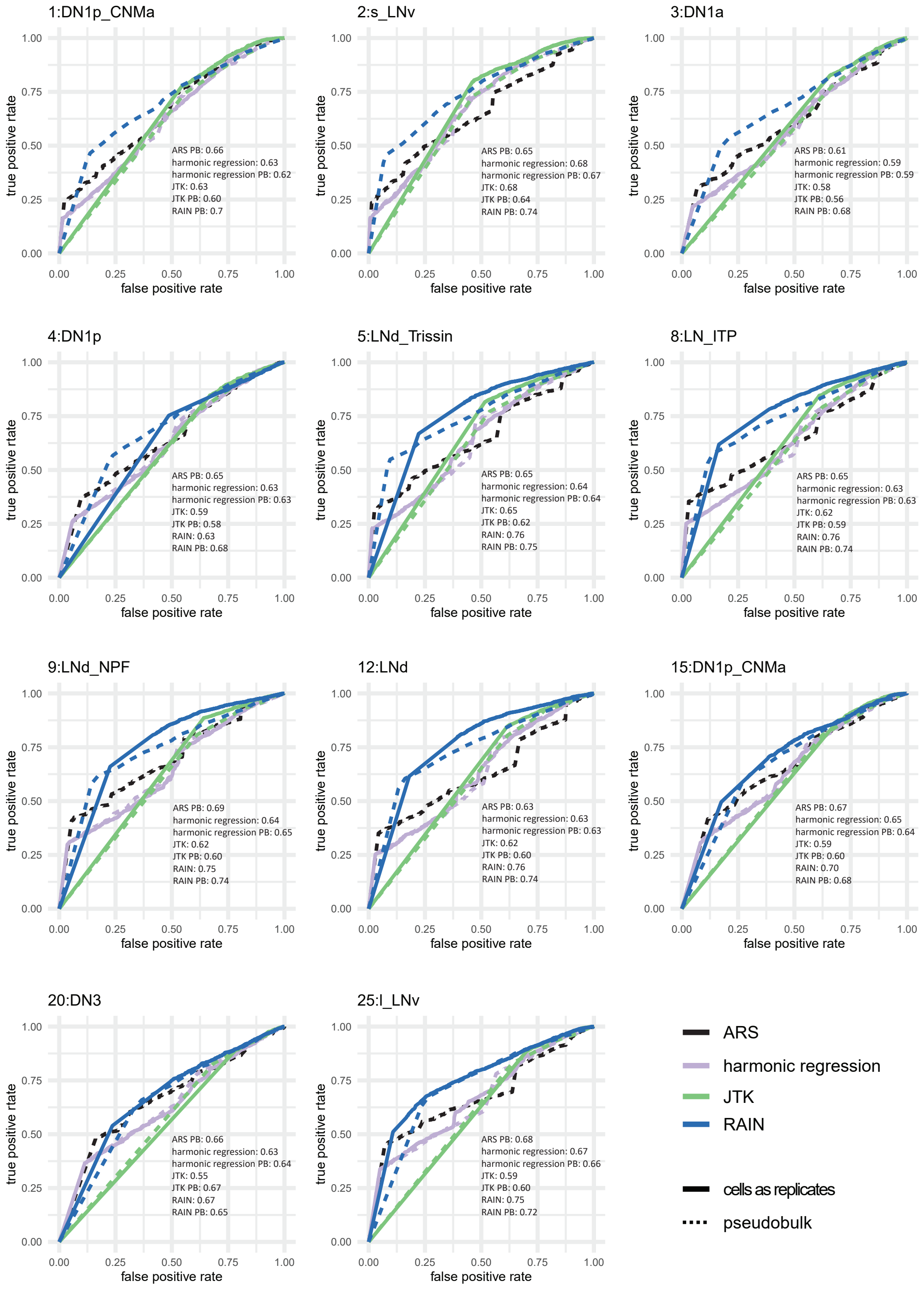

Figure S9: ROC curves of predicting circadian genes in bulk tissue using single cell RNA-seq data. All cells are supplied to the cycling detection algorithms and p-values are used for cycling prediction. l_LNv and s_LNv neurons were compared to bulk samples collected from the ventral lateral neurons. DN1p, DN1a neurons were compared to bulk samples collected from the dorsal neurons. LND neurons were compred to bulk samples collected from the dorsal lateral neurons. LN_ITP neurons were compared to samples taken from both the doral and ventral lateral neurons.
