# Supplementary material for: Detecting Rhythmic Gene Expression in Single Cell Transcriptomics
Source: bioRxiv. 2023 Dec 8:2023.12.07.570691. Preprint. [Version 1] doi: 10.1101/2023.12.07.570691 (PMC10723455; doi:10.1101/2023.12.07.570691)
Supplement: Supplementary file 1 [file NIHPP2023.12.07.570691V1-supplement-1.pdf]

bioRxiv preprint doi: https://doi.org/10.1101/2023.12.07.570691; this version posted December 8, 2023. The copyright holder for this preprint (which was not certified by peer review) is the author/funder, who has granted bioRxiv a license to display the preprint in perpetuity. It is made available under aCC-BY 4.0 International license.

6 Supplemental material

6.1 Data preprocessing and clustering

Preprocessed data and cell type information was contained within the Seurat object provided by Ma et al [23]. Single cell data from six time points (two experimental replicates, hence twelve in total) were processed by Ma et al., who integrated the data on a per time point basis using Seurat with genes that identified by Seurat as highly variable across all time points. Integrated data were then reduced to 2D using t-SNE, from which cluster identities were assigned. For all of our analysis, we used normalized counts kindly shared my Ma et al. to ensure correspondence with results reported in [23].

6.2 Cycling detection

Given the two experimental replicates from Ma et al., were collected consecutively, the data were concatenated to a time series with twelve time points. Consequently, the pseudobulked time series also consist of twelve data points, and similarly for the subsampled data (constructed such that each subexperiment has at least one sample from each one of the twelve time points).

We applied four cycling detection methods:

- JTK-cycle is implemented using $JTK\_CYCLEv3.1.R$. The period was set to 6 and the sampling interval was set to 4 to match the experimental procedure. When considering single cells as replicates, we also supplied the number of samples for each time point.
- Harmonic regression is implemented using the harmonicRegression package in R [27] inputting only time and the expression matrix.
- RAIN is implemented in the R package [18]. It takes as input the expression matrix ordered by sampling time and the sampling interval *deltat*, set to 4 to match the experimental design.
- ARSER is implemented using the metacycle package in R [26], whose only required input is the expression matrix and sampling time.

For the purpose of our analysis, a gene is considered to be cycling if its raw p-value is less than 0.05. We also repeat the analysis using FDR–adjusted p-values.

6.3 Derivation of theoretical computational complexity

6.3.1 Mann-Whitney U Statistics

Both JTK cycle and RAIN are non-parametric methods that are built upon the Mann-Whitney U statistics. Let $(X_{11}, \ldots, X_{1m})$,... $(X_{T1}, \ldots, X_{Tm})$ be a set of T time–associated observations following probability distributions $P_1(x)$, ..., $P_T(x)$ for timepoints 1–T. For the purpose of deriving computational complexity, we have assumed that each time point contains the same number of cells m without loss of generality. The Mann-Whitney test tests the null hypothesis that $P_i = P_j (i \neq j)$ by computing the U statistic between sample i and sample j as

$$U_{i,j} = \sum_{k=1}^{m} \sum_{l=1}^{m} \mathbb{I}(X_{ik} < X_{jl}) \tag{3}$$

It is easy enough to see that $U_{i,j}$ should have a computational complexity of $\mathcal{O}(m^2)$ and therefore the computational complexity of $\mathbf{U} = (U_{1,2}, \ldots, U_{T-1,T})$ should be approximately $\mathcal{O}(T^2 \times m^2)$. Since in real datasets $T \ll m$ and the number of time points do not change from one cluster to another, our expected observed computational complexity, when changing sample size, should also be $\mathcal{O}(m^2)$.

6.3.2 Jonckheere-Terpstra Test

JTK cycle employs the Jonckheere-Terpstra test, which is an extension of the Mann-Whitney U statistics to the case of having more than two samples. It tests for the presence of monotonic trend with the test statistics s, defined as

$$s = \sum_{i=1}^{T-1} \sum_{j=i+1}^{T} U_{i,j} \tag{4}$$

bioRxiv preprint doi: https://doi.org/10.1101/2023.12.07.570691; this version posted December 8, 2023. The copyright holder for this preprint (which was not certified by peer review) is the author/funder, who has granted bioRxiv a license to display the preprint in perpetuity. It is made available under aCC-BY 4.0 International license.

Again, it is clear that the computational complexity for s should be $\mathcal{O}(m^2 + T^2)$. When the number of time points is fixed, the computational complexity scales quadratically with sample size.

In JTK cycle, the rising and falling part of the oscillation is tested for each a pre-determined waveform (e.g., sine waves of differing phases), resulting in a complexity of $\mathcal{O}(km^2)$, where k is the number of templates. As k is fixed and $k \ll m$, the complexity will scale approximately $\mathcal{O}(m^2)$.

6.3.3 General Umbrella

To overcome the JTK-cycle's loss of power when considering multiple waveforms, RAIN uses a variation of the general umbrella which tests for the presence of an umbrella shape, which can be formally written as

$$H1 : P_1(x) < P_2(x) < \dots P_t(x) > \cdots > P_T(x) \tag{5}$$

Here, t is a predetermined inflection point. The test statistics of the general umbrella is the sum of two Jonckheere-Terpstra statistics

$$s = \sum_{i=1}^{t-1} \sum_{j=i+1}^{t} U_{i,j} + \sum_{i=t}^{T-1} \sum_{j=i+1}^{T} U_{j,i} \tag{6}$$

RAIN tests for all potential inflection points T, yielding a computational complexity $\mathcal{O}(Tm^2)$. Since T is fixed by the experimental design, the complexity scales with the squared number of cells.

bioRxiv preprint doi: https://doi.org/10.1101/2023.12.07.570691; this version posted December 8, 2023. The copyright holder for this preprint (which was not certified by peer review) is the author/funder, who has granted bioRxiv a license to display the preprint in perpetuity. It is made available under aCC-BY 4.0 International license.

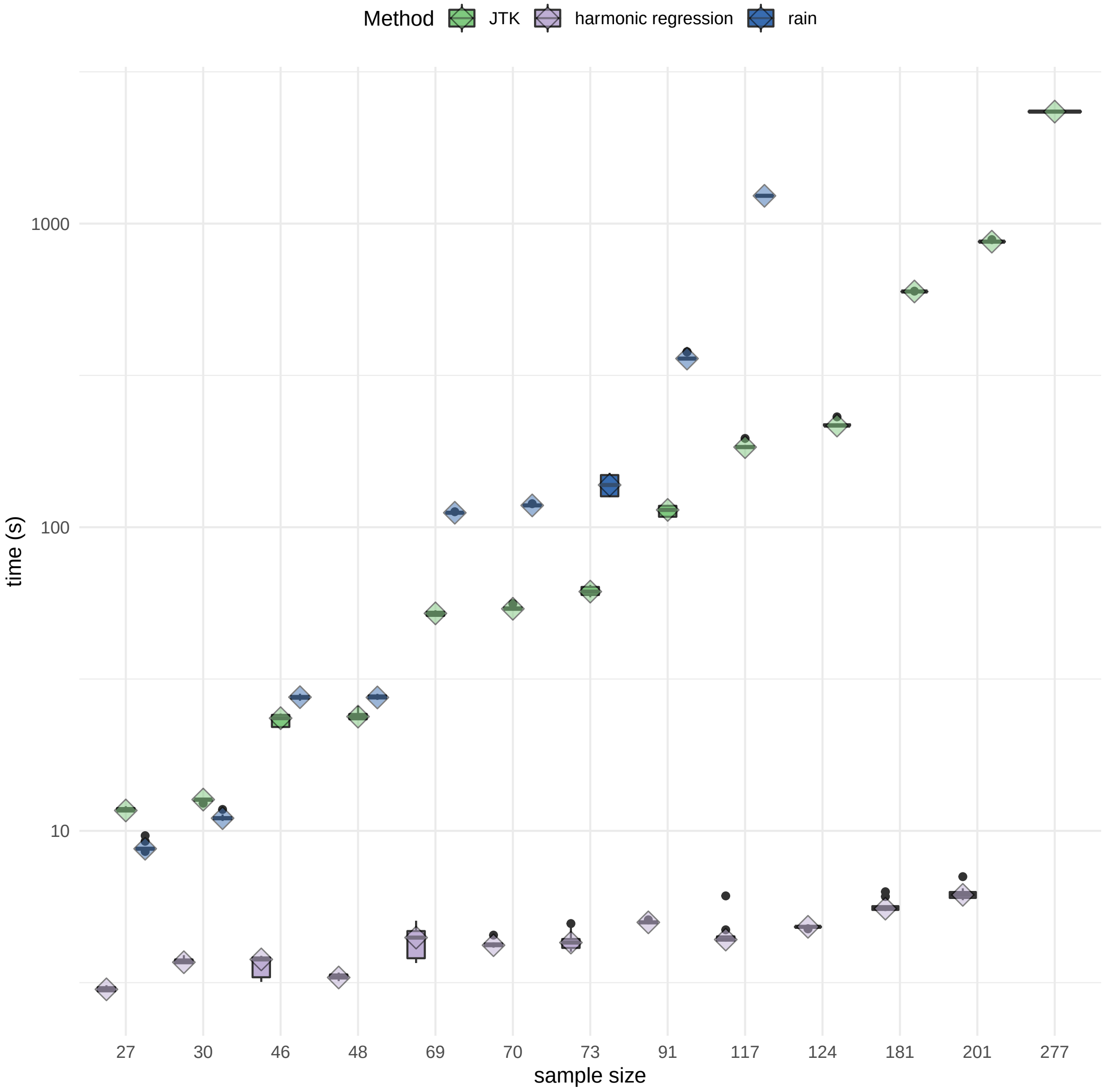

Figure S1: Run time for JTK cycle, RAIN and harmonic regression on multiple cell types.

bioRxiv preprint doi: https://doi.org/10.1101/2023.12.07.570691; this version posted December 8, 2023. The copyright holder for this preprint (which was not certified by peer review) is the author/funder, who has granted bioRxiv a license to display the preprint in perpetuity. It is made available under aCC-BY 4.0 International license.

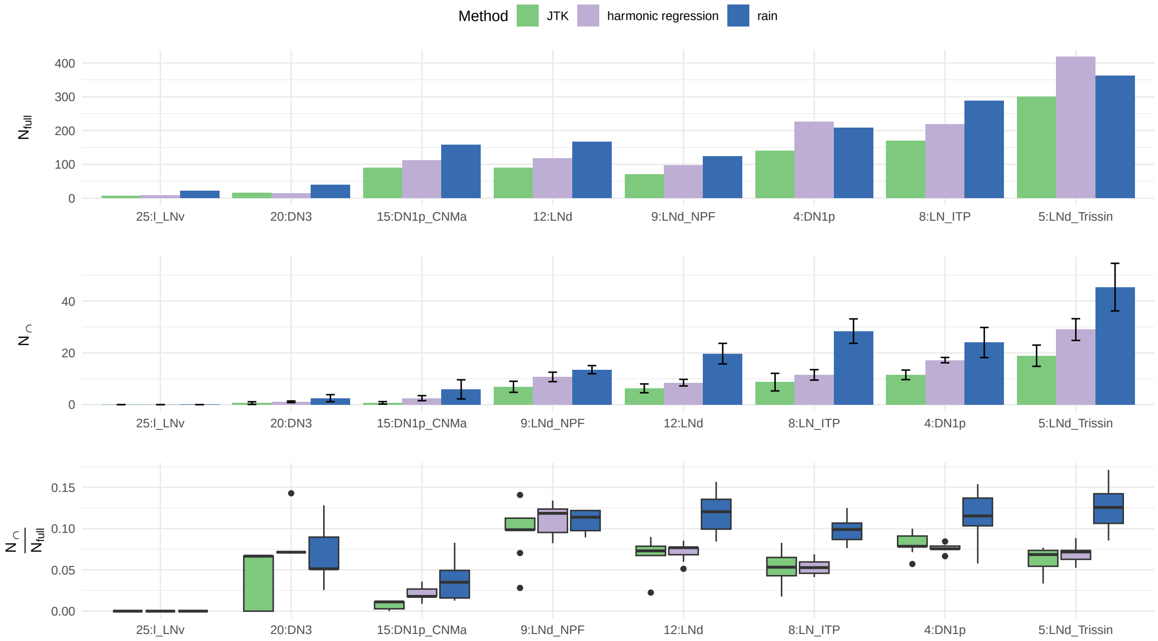

Figure S2: Top: The number of cycling genes detected using all cells, N_{full}. Middle: The average number of cyclers detected in both subsamples across 10 trials, $N_\cap$. Bottom: The ratio of $N_\cap$ to N_{full}, the proportion of consistently–detected sub-sample cyclers relative to those found using all cells. Error bars indicate standard deviation across the 10 subsamplings. A gene is considered cycling if its BH corrected p-value (i.e., FDR) is less than 0.05.

bioRxiv preprint doi: https://doi.org/10.1101/2023.12.07.570691; this version posted December 8, 2023. The copyright holder for this preprint (which was not certified by peer review) is the author/funder, who has granted bioRxiv a license to display the preprint in perpetuity. It is made available under aCC-BY 4.0 International license.

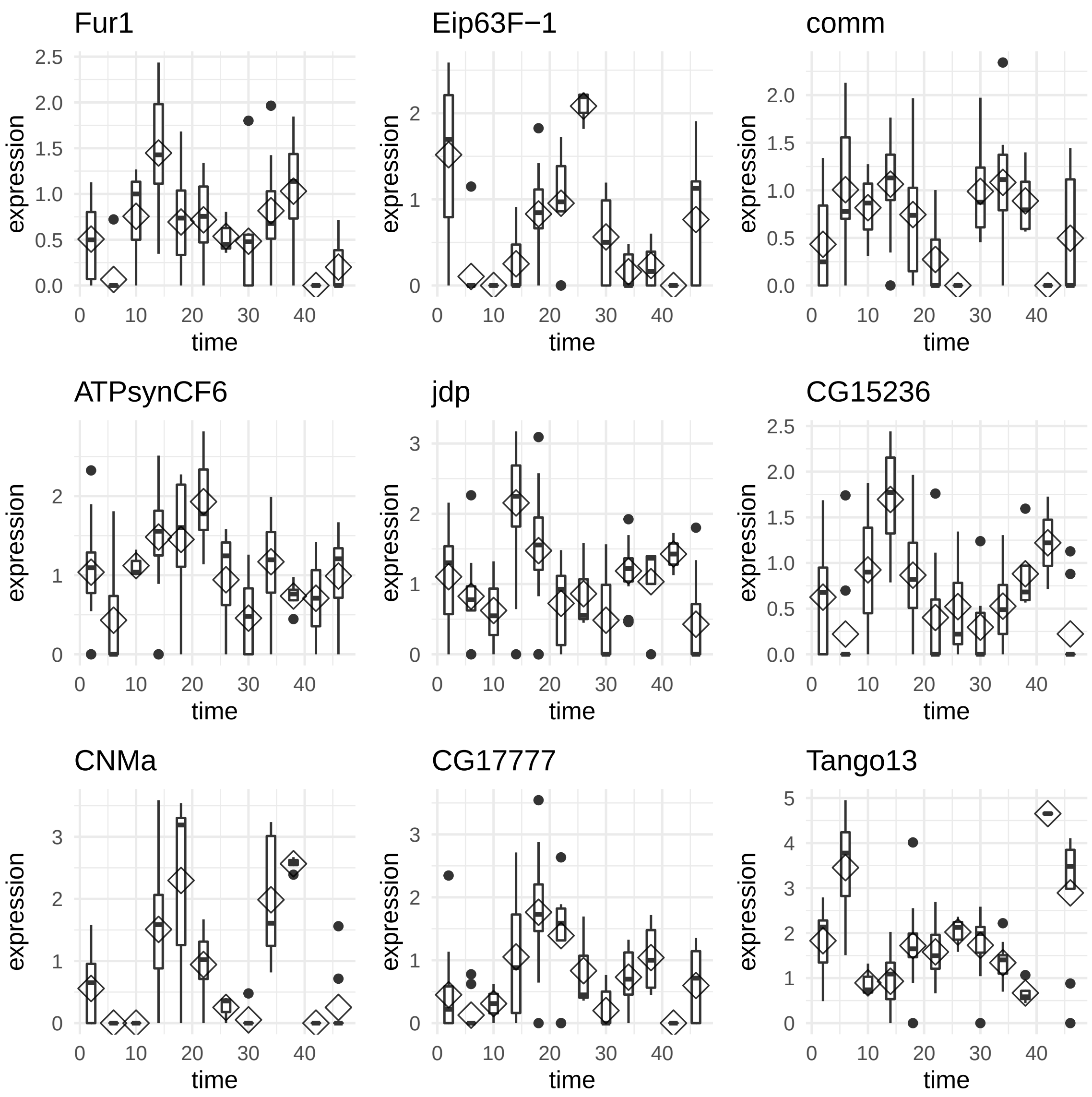

Figure S3: Example genes considered cycling by JTK-cycle when all cells were used as replicates but not when pseudobulk expression was used.

bioRxiv preprint doi: https://doi.org/10.1101/2023.12.07.570691; this version posted December 8, 2023. The copyright holder for this preprint (which was not certified by peer review) is the author/funder, who has granted bioRxiv a license to display the preprint in perpetuity. It is made available under aCC-BY 4.0 International license.

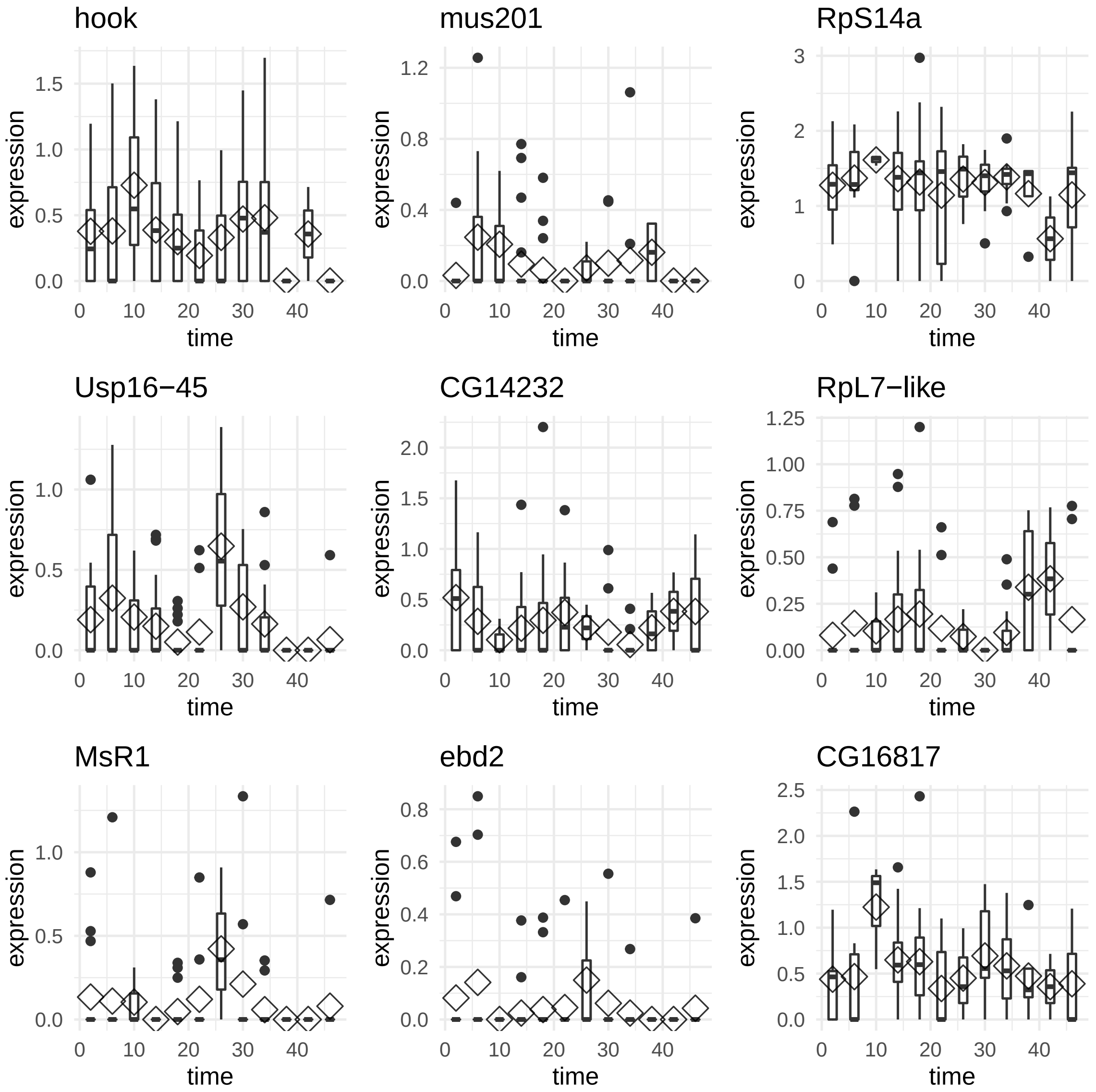

Figure S4: Example genes considered cycling by JTK cycle using pseudobulk expression but not when cells were treated as replicates

bioRxiv preprint doi: https://doi.org/10.1101/2023.12.07.570691; this version posted December 8, 2023. The copyright holder for this preprint (which was not certified by peer review) is the author/funder, who has granted bioRxiv a license to display the preprint in perpetuity. It is made available under aCC-BY 4.0 International license.

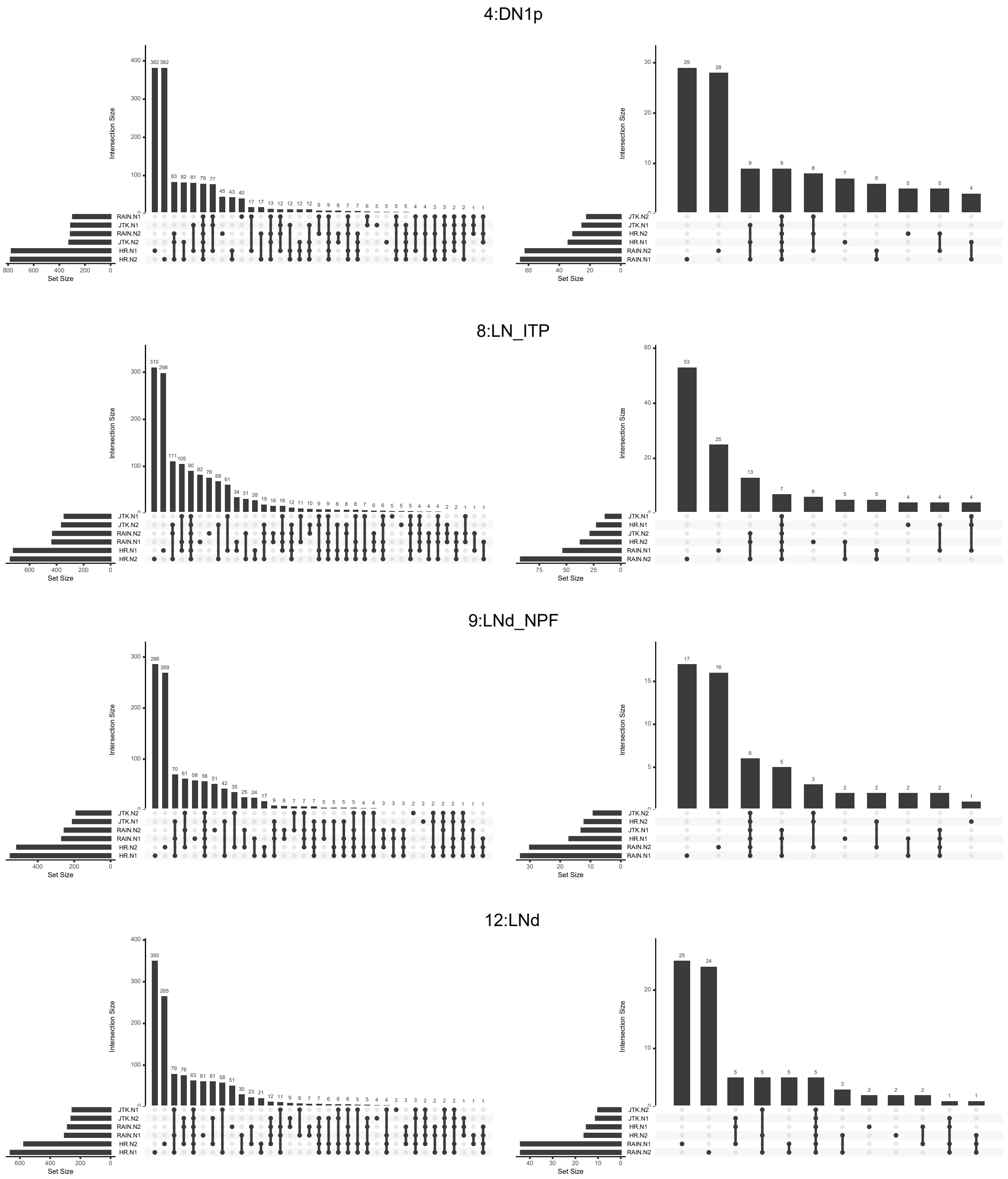

Figure S5: Upset plot of cluster 4, 8, 9, and 12. The left (right) column uses p-values (adjusted p-values) to identify cycling genes.

bioRxiv preprint doi: https://doi.org/10.1101/2023.12.07.570691; this version posted December 8, 2023. The copyright holder for this preprint (which was not certified by peer review) is the author/funder, who has granted bioRxiv a license to display the preprint in perpetuity. It is made available under aCC-BY 4.0 International license.

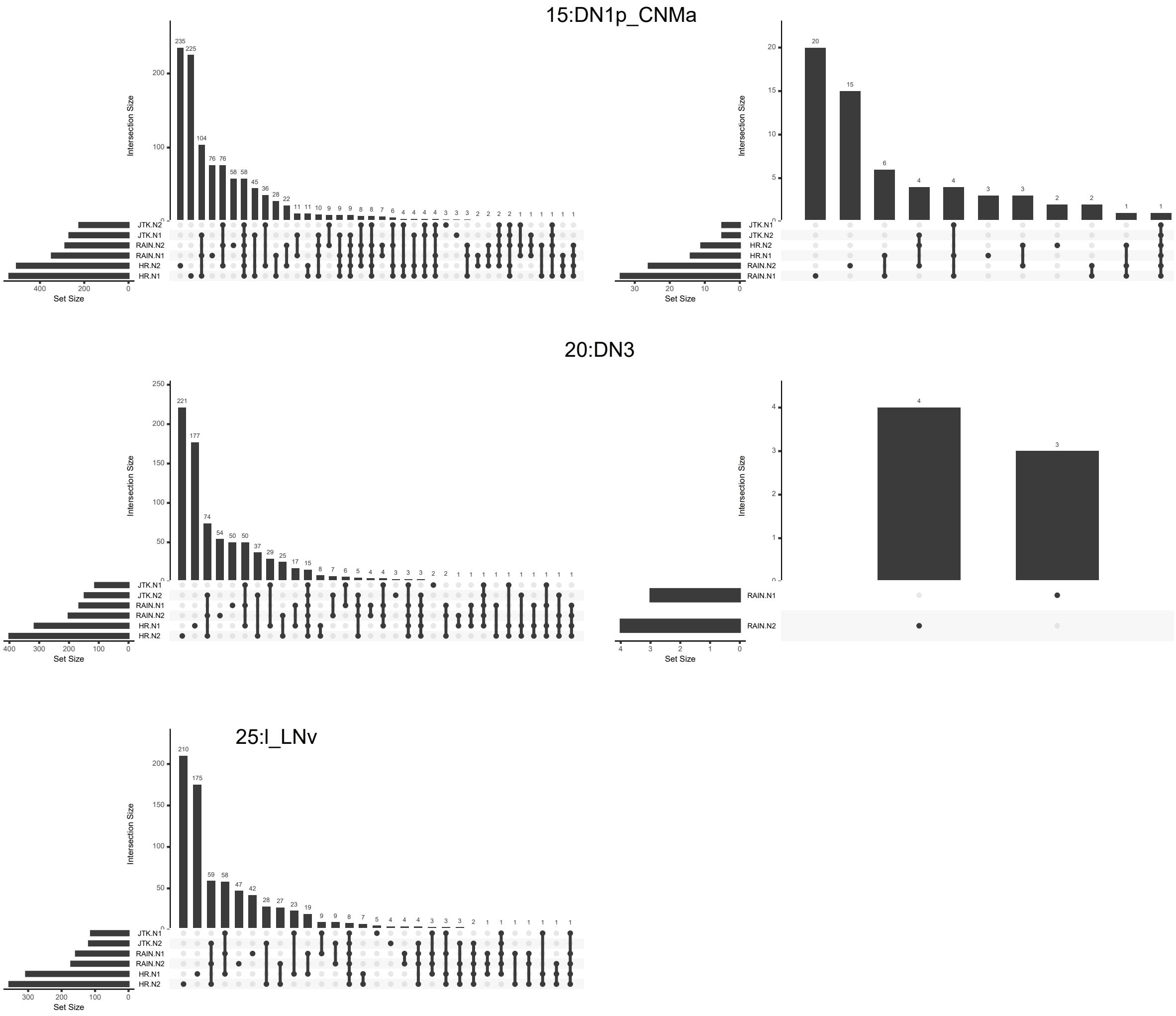

Figure S6: Upset plot of cluster 15, 20, and 25. The left (right) column uses p-value (adjusted p-values) to identify cycling genes. Right column for cluster 25 is empty for that the average intersection between all sets were less than one.

bioRxiv preprint doi: https://doi.org/10.1101/2023.12.07.570691; this version posted December 8, 2023. The copyright holder for this preprint (which was not certified by peer review) is the author/funder, who has granted bioRxiv a license to display the preprint in perpetuity. It is made available under aCC-BY 4.0 International license.

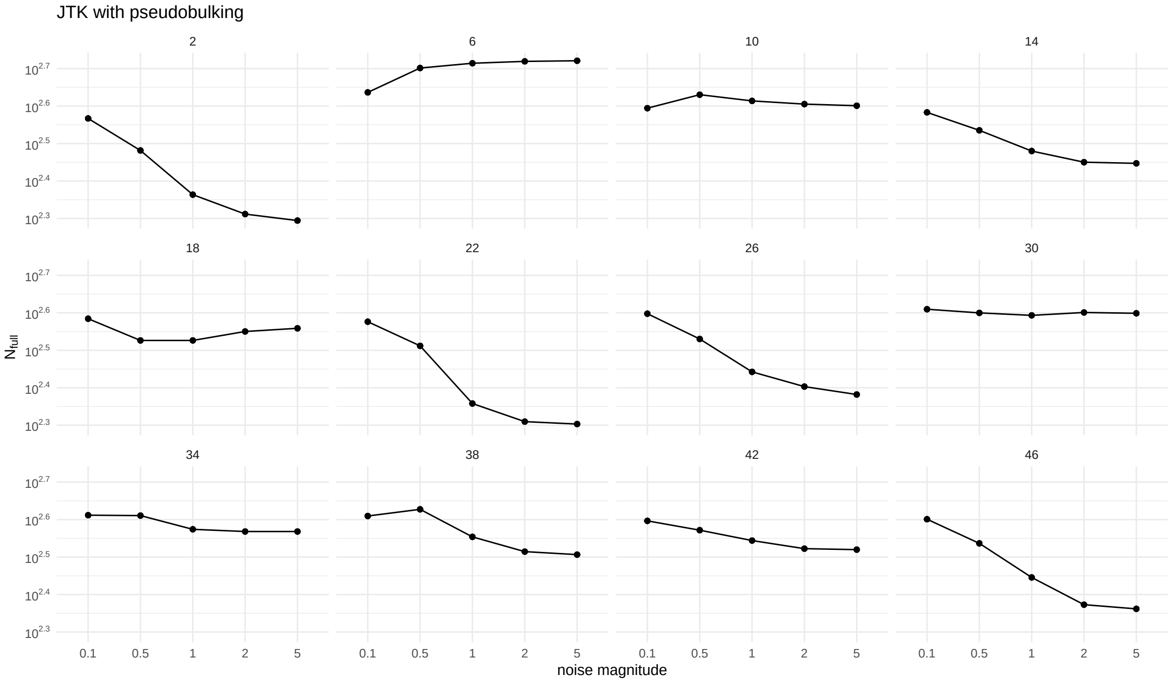

Figure S7: Effect of systematic contamination when using JTK-cycle with pseudobulking. Each panel shows how the number of detected cyclers N_{full} varies as function of the magnitude of noise injected at a single time-point.

bioRxiv preprint doi: https://doi.org/10.1101/2023.12.07.570691; this version posted December 8, 2023. The copyright holder for this preprint (which was not certified by peer review) is the author/funder, who has granted bioRxiv a license to display the preprint in perpetuity. It is made available under aCC-BY 4.0 International license.

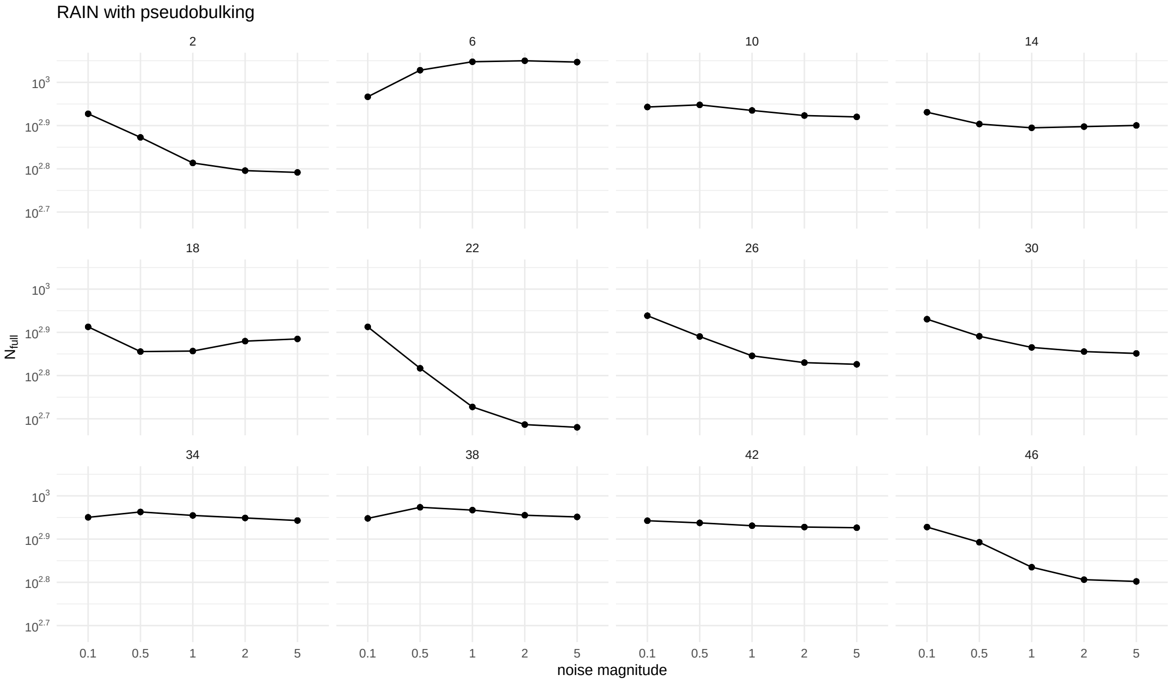

Figure S8: Effect of systematic contamination when using RAIN with pseudobulking. Each panel shows how the number of detected cyclers N_{full} varies as function of the magnitude of noise injected at a single time-point.

bioRxiv preprint doi: https://doi.org/10.1101/2023.12.07.570691; this version posted December 8, 2023. The copyright holder for this preprint (which was not certified by peer review) is the author/funder, who has granted bioRxiv a license to display the preprint in perpetuity. It is made available under aCC-BY 4.0 International license.

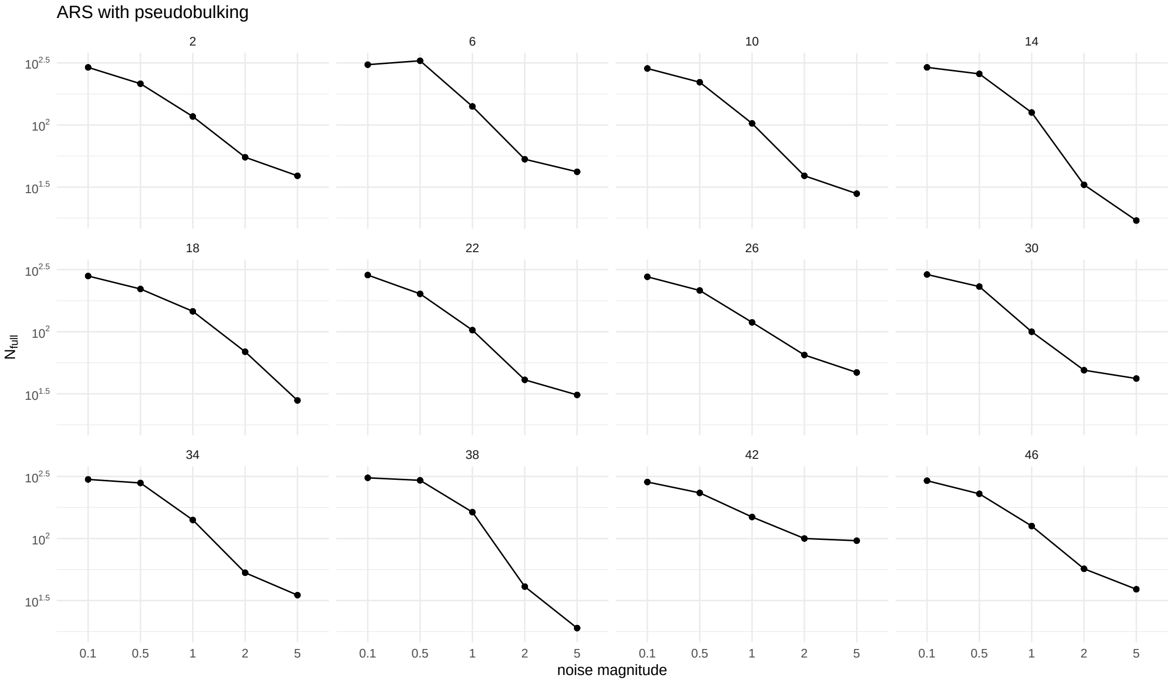

Figure S9: Effect of systematic contamination when using ARSER with pseudobulking. Each panel shows how the number of detected cyclers N_{full} varies as function of the magnitude of noise injected at a single time-point. The time-point contaminated by noise is given at the top of each panel.

bioRxiv preprint doi: https://doi.org/10.1101/2023.12.07.570691; this version posted December 8, 2023. The copyright holder for this preprint (which was not certified by peer review) is the author/funder, who has granted bioRxiv a license to display the preprint in perpetuity. It is made available under aCC-BY 4.0 International license.

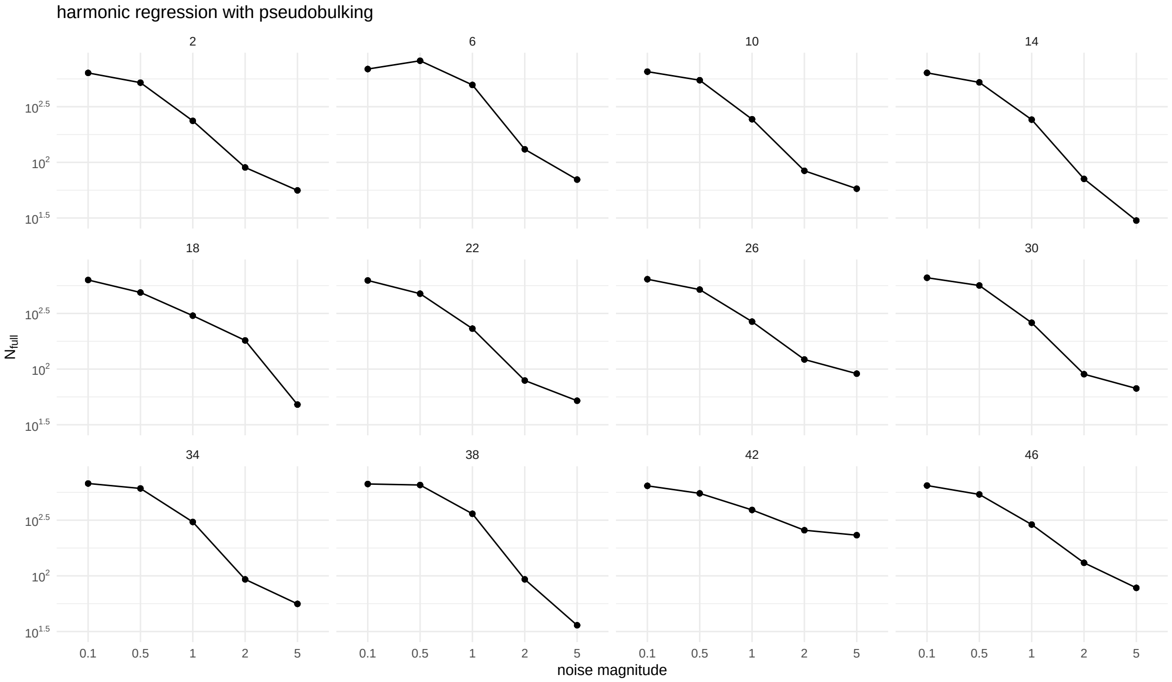

Figure S10: Effect of systematic contamination when using harmonic regression with pseudobulking. Each panel shows how the number of detected cyclers N_{full} varies as function of the magnitude of noise injected at a single time-point. The time-point contaminated by noise is given at the top of each panel.

bioRxiv preprint doi: https://doi.org/10.1101/2023.12.07.570691; this version posted December 8, 2023. The copyright holder for this preprint (which was not certified by peer review) is the author/funder, who has granted bioRxiv a license to display the preprint in perpetuity. It is made available under aCC-BY 4.0 International license.

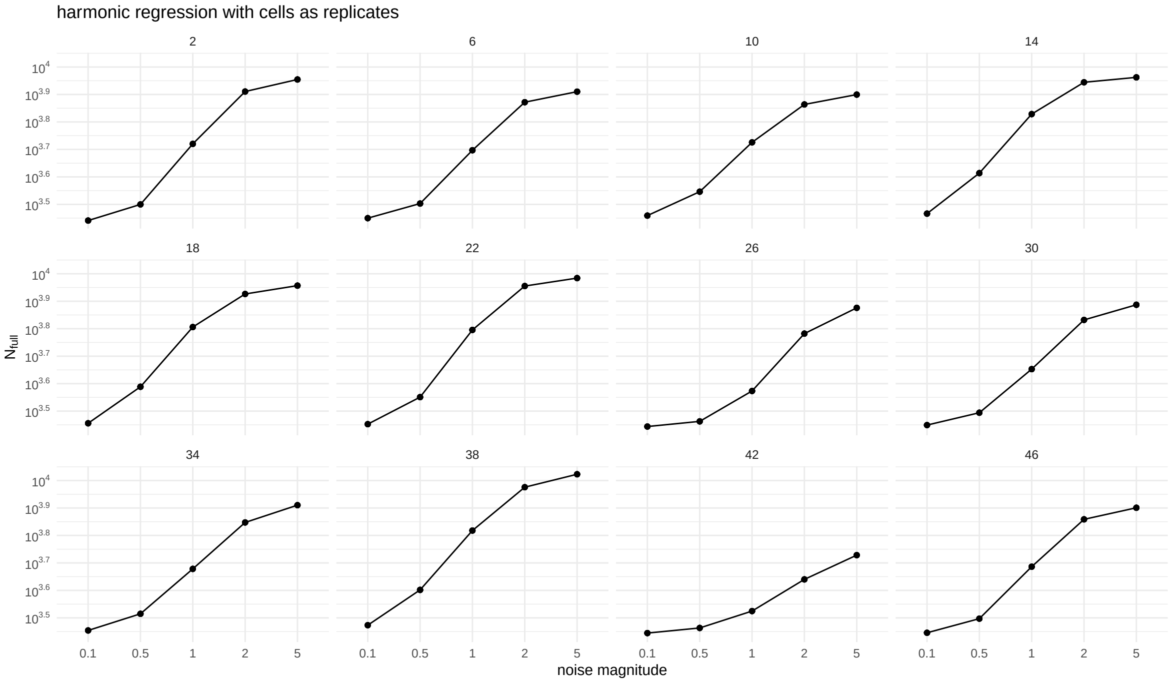

Figure S11: Effect of systematic contamination when using harmonic regression with cells as replicates. Each panel shows how the number of detected cyclers N_{full} varies as function of the magnitude of noise injected at a single time-point. The time-point contaminated by noise is given at the top of each panel.

bioRxiv preprint doi: https://doi.org/10.1101/2023.12.07.570691; this version posted December 8, 2023. The copyright holder for this preprint (which was not certified by peer review) is the author/funder, who has granted bioRxiv a license to display the preprint in perpetuity. It is made available under aCC-BY 4.0 International license.

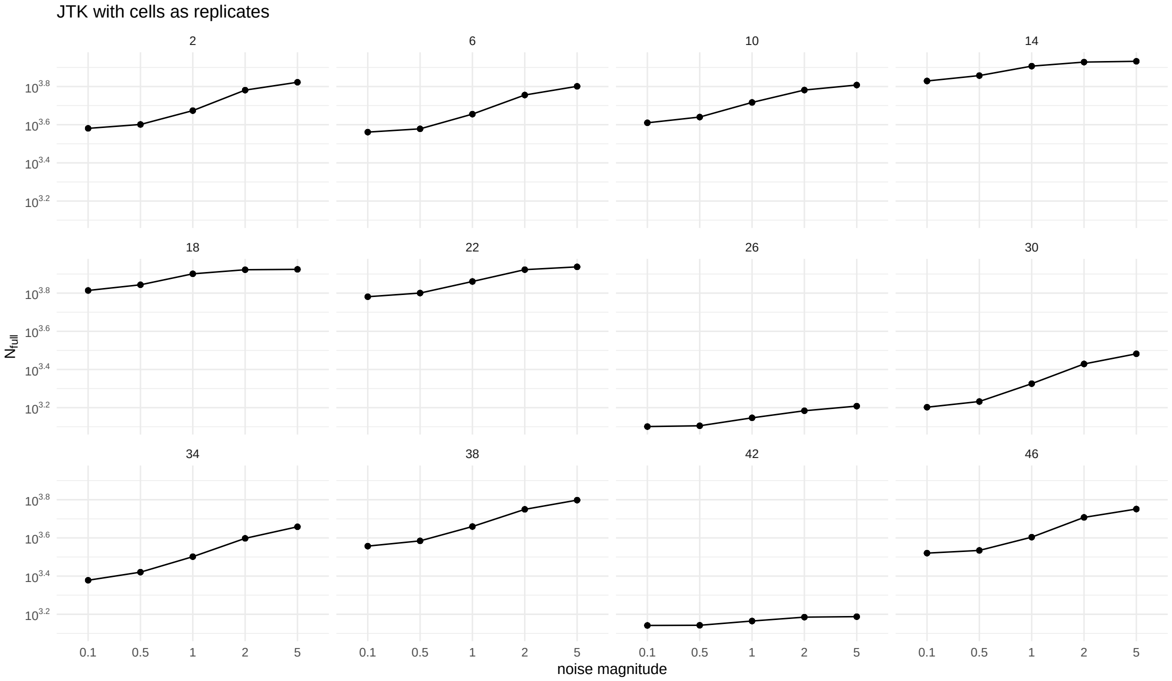

Figure S12: Effect of systematic contamination when using JTK-cycle with cells as replicates. Each panel shows how the number of detected cyclers $N_{\rm full}$ varies as function of the magnitude of noise injected at a single time-point. The time-point contaminated by noise is given at the top of each panel.
